# Optimization of Lactic Acid Production from Pineapple By-Products and an Inexpensive Nitrogen Source Using *Lactiplantibacillus plantarum* strain 4O8

**DOI:** 10.1155/2021/1742018

**Published:** 2021-10-19

**Authors:** Joel Romial Ngouénam, Pierre Marie Kaktcham, Chancel Hector Momo Kenfack, Edith Marius Foko Kouam, François Zambou Ngoufack

**Affiliations:** Research Unit of Biochemistry, Medicinal Plants, Food Science and Nutrition (URBPMAN) Department of Biochemistry, Faculty of Science, University of Dschang, P.O. Box 67, Dschang, Cameroon

## Abstract

Lactic acid (LA) is used in food, cosmetic, chemical, and pharmaceutical industries and has recently attracted much attention in the production of biodegradable polymers. The expensive substances including carbon and nitrogen sources involved in its fermentative synthesis and the increasing market demand of LA have prompted scientists to look for inexpensive raw materials from which it can be produced. This research was aimed at determining the optimum conditions of lactic acid (LA) production from pineapple by-products and an inexpensive nitrogen source using *Lactiplantibacillus plantarum* strain 4O8. After collection and preparation of the carbon source (pineapple by-products) and nitrogen sources (by-products from fish, chicken, and beer brewing industries), they were used for the formulation of 4 different media in terms of nitrogen sources. Then, the proximate compositions of promising nitrogen sources were determined. This was followed by the screening of factors (temperature, carbon source, nitrogen source, MgSO_4_, MnSO_4_, FeSO_4_, KH_2_PO_4_, and KHPO_4_) influencing the production of LA using the definitive plan. Lastly, the optimization process was done using the central composite design. The highest LA productions (14.64 ± 0.05 g/l and 13.4 ± 0.02 g/l) were obtained in production medium supplemented with chicken and fish by-products, respectively, making them the most promising sources of nitrogen. The proximate analysis of these nitrogen sources revealed that their protein contents were 83.00 ± 1.41% DM and 74.00 ± 1.41% DM for chicken by-products and fish by-products, respectively. Concerning the screening of factors, temperature, nitrogen source, and carbon source were the factors that showed a major impact on LA production in the production medium containing chicken by-products as nitrogen source. A pineapple by-product concentration of 141.75 g/l, a nitrogen source volume of 108.99 ml/l, and a temperature of 30.89°C were recorded as the optimum conditions for LA production. The optimization led to a 2.73-fold increase in LA production when compared with the production medium without nitrogen source. According to these results, chicken by-products are a promising and an inexpensive nitrogen source that can be an alternative to yeast extract in lactic acid production.

## 1. Introduction

Many substances which contribute to human well-being result from microorganism's exploitation; LA is concerned [1]. It is an important chemical that can be produced by lactic acid bacteria for many potential applications. LA has applications in pharmaceutical (dialysis solution, mineral preparations, tableting, surgical sutures, and prosthesis), chemical (pH regulators, chiral intermediates, cleaning agent, metal complexing agents, and green solvent), food (acidulant, flavor enhancer, preservatives, mineral fortification, and antioxidant), and cosmetic (antitartar agents, antiacne agents moisturizers, skin-lightening agents, skin rejuvenating agents, and humectants); it is also used in the biodegradables polymers production [2, 3]. The global LA market is not insignificant; indeed, it will increase from 1220.0 kilotons in 2016 to 1960.1 kilotons in 2025 and represent a turnover of about 9.8 billion dollars [3]. LA can be produced by chemical synthesis or fermentation. In recent years, the fermentation approach has become more successful; 95% of industrial production of LA is obtained by fermentation pathway since this natural way generates optically pure lactate, and also, by-products from this process do not pollute the environment [4–7]. However, this natural way of producing LA has a real production cost problem caused by the use of expensive substrates, especially carbon sources (glucose, sucrose, lactose, and maltose) and also nitrogen sources (yeast extract, ammonium sulphate, and peptone) [4]. Furthermore, the availability of LA and also its accessibility for the various applications in the different aforementioned industries would thus be limited. It is therefore essential to search for alternatives to these expensive substrates. Indeed, the production medium plays an important part in the cost of producing biomass, metabolites, and therefore LA; it is consequently essential to find a balance between the nutritional and the technological qualities of the raw materials, their prices, and their availability. Thus, by-products from the food industry with regard to their carbohydrate and protein composition [4, 8–10] could contribute to reducing the costs involved in the production of lactic acid by the fermentation way. Several scientific studies have focused on the use of by-products from food industry as a carbon source (molasses, fruit by-products, whey, cassava bagasse, starch, sweet sorghum juice, and lignocellulose biomass) [3, 7, 11–15] and also as a nitrogen source (corn steep liquor, malt buds, red lentil, and baker's yeast cells) for the LA production [4, 16, 17]. However, the use of by-products from the processing and marketing chain of fish, chicken, and beer industries production as a nitrogen source in a production medium containing pineapple by-products as a carbon source is still unknown to the scientific community.

The objective of the present study was to determine the optimum conditions of lactic acid production from pineapple by-products and an inexpensive nitrogen source using *Lactobacillus plantarum* 4O8 strain.

## 2. Materials and Methods

### 2.1. Bacterial Strain, Media, and Growth Conditions

The *Lactiplantibacillus plantarum* strain 4O8 used in this study was obtained from our research unit collection. This strain was isolated from orange sold in Dschang city during our previous studies [10] and identified by 16S rRNA gene sequencing and its sequence deposited in the NCBI GenBank under the accession number MW164849. This strain was selected as a good fermenter of pineapple by-products. The reactivation (1% *v*/*v*) of the strain was carried out in the previously sterilized MRS broth (TM MEDIA, Titan Biotech Ltd, India) medium from the stored culture which was kept at -20°C in the reconstituted skim milk (12.5% (*w*/*v*) supplemented with 25% (*v*/*v*) glycerol [18]. After 12 hours of growth at 30°C, the strain was used for further work.

### 2.2. Preparation and Chemical Composition of Carbon Source

#### 2.2.1. Chemical Composition of Carbon Source

Previous work [10] showed the chemical proximate composition of pineapple by-products (carbon source).

#### 2.2.2. Preparation of Carbon Source

Pineapple by-products were collected at the central market of the city of Dschang and sent to the laboratory for further processing. The modified method of Mudaliyar et al. [11] was used for the preparation of carbon sources. Five hundred grams (500 g) of these substrates was steam treated in an autoclave (Sonoclav) at 121°C for 20 minutes. Once cooled, distilled water was added to our substrate to make a volume of 1 l and was then boiled at 80°C for 30 minutes in a water bath (HH-W420, China). Finally, the hydrolysate was recovered by filtration using a cotton veil and acid hydrolysis of the filtrate obtained previously was carried out by autoclaving it at 121°C with 1% (*v*/*v*) 2 M HCl for 20 min. The pH of the hydrolysate obtained was adjusted to a value of 6.6 with CaO, and the CaSO_4_ precipitate formed was removed by filtration with Whatman filter paper No.1.

### 2.3. Preparation and Proximate Analysis of Nitrogen Sources

#### 2.3.1. Preparation of Nitrogen Sources

*(1) Source of Nitrogen from Chicken (BC) and Fish (BF)*. Nitrogen sources were prepared following the method described by Ben et al. [19] with modifications. The fish by-products consisted of heads, viscera, bones, and muscle residues. Concerning the chicken by-products, they consisted of chicken intestines, pancreas, heart-lung, and their heads. All these nitrogen sources were aseptically collected in sterile coolers in fish shops and poultry slaughterhouses of the central market of the city of Dschang (Menoua Division, West Cameroon region). They were transported to the laboratory where they were subjected to various treatments. After cleaning, fish and chicken by-products were minced by a blender, mixed with sterile water (500 : 1 g/1), and heated at 100°C for 20 minutes. After this heat pretreatment, the insoluble material was removed by centrifugation (4500 rpm for 30 min), and the supernatant (BF and BC) was collected and kept at 4°C until further use.

*(2) Source of Nitrogen from the Beer Production Industry (BBIP)*. In the beer production industries in Cameroon, the dead cells of *Saccharomyces cerevisiae* resulting from the fermentation and which constitute a source of protein [20] are mixed with dregs to enrich it before being made available to the various users. Dregs, a by-product of these industries, were aseptically collected in sterile coolers in the Cameroon Brewery company of the city of Bafoussam (Mifi Division, West Cameroon region) and transported to the laboratory. The preparation of the nitrogen source from this by-product was carried out as described by Zarei et al. [20]. Five hundred grams (500 g) of this by-product was introduced into 2 l of distilled water, and then the whole was heat treated by autoclaving (121°C, 15 min, 1.2 bar) and followed by quenching in an ice bath. The insoluble material was then removed by centrifugation (4500 rpm for 30 min), and the supernatant was collected. Finally, the previously collected supernatant was again subjected to the two treatments mentioned above (autoclaving and centrifugation), and the new supernatant (BBIP) was collected and kept at 4°C until further use.

#### 2.3.2. Proximate Analysis of Nitrogen Sources

According to the methods described by IUPAC [21] and AOAC [22], the proximate composition (fat, protein, ash, P, Ca, Mg, Na, Mn, Fe, Zn, and K) of nitrogen sources was assessed.

### 2.4. Preparation of the Inoculum and Fermentation Conditions

The modified method of Herdian et al. [23] was used for the preparation of the inoculum. Young (12 h-old cultures) cultures were streaked on previously cast and solidified MRS agar. The inoculated medium was incubated for 48 h. Then, the bacteria were aseptically collected from the surface of the agar and suspended in 10 ml of a sterile NaCl solution (0.9%). The mixture was then vortex agitated and its opacity adjusted to McFarland's scale 2. The cell suspension corresponding to McFarland scale 2 (approximately 6 × 10^8^ CFU/ml) was used as inoculum for the fermentation.

Batch cultures were carried out in 250 ml Erlenmeyer units with a useful volume of 100 ml of the supplemented and sterile hydrolysate. The synthetic medium for the fermentation was prepared using Mudaliyar et al.'s [11] method with modification: hydrolysate: 1000 ml; nitrogen: 60 ml; sodium acetate: 5 g; MgSO_4_.7H_2_0: 0.6 g; MnSO_4_.H_2_0: 0.05 g; K_2_HPO_4_: 0.8 g; KH_2_PO_4_: 0.8 g; and FeSO_4_: 0.05 g. Then, this fermentation medium was inoculated at a rate of 5% (*v*/*v*) and incubated at 30°C under agitation after every 2 h for 2 days. Substrate consumption and lactic acid production were determined on a regular time range (0, 8, 16, 24, 32, and 48 h).

### 2.5. Estimation of Reducing Sugars Consumption and Lactic Acid Production in the Production Medium

Using DNS (3,5-dinitro salicylic acid) method as described by Fisher and Stein [24], the residual reducing sugar content of the production medium was estimated on regular basis spectrophotometrically. The results obtained were the mean of three experiments.

The spectrophotometric method described by Borshchevskaya et al. [25] was used for lactic acid quantification. One ml of the fermentation broth was centrifuged (4500 rpm for 30 min), and the supernatant was collected and diluted 10 times. Subsequently, 0.1 ml of the previous dilution was added to 4 ml of a FeCl3 solution (0.2%); after homogenization, the optical density was measured with a spectrophotometer (Thermo Scientific BioMate 3S UV-Visibles spectrophotometer, Thermo Scientific, USA) at a wavelength of 390 nm. Based on a previously drawn calibration curve, the amount of lactic acid was calculated. The experiments were repeated three times.

### 2.6. Screening for the most Significant Factors Influencing LA Production by Definitive Design

A definitive plan was used for the screening of factors influencing LA production. Based on the literature and previous works, 8 factors were chosen including nitrogen source, carbon source, temperature, magnesium sulphate, dipotassium phosphate, monopotassium phosphate, manganese sulphate, and ferrous sulphate; the time was kept constant (16 h). The intervals of these different factors are summarized in Table 1. A total number of 17 experimental runs (Table 2) were obtained, and LA production was used as response. The number of tests was determined by applying the formula 2*k* + 1 with *k* representing the number of factors. The result obtained for each run was the mean of three experiments.

### 2.7. Optimization of LA Production by Central Composite Design

The central composite design (CCD) with 3 independent variables (nitrogen source, temperature, and carbon source) was used to determine the optimal conditions for the production of LA; indeed, these factors are those that have previously shown a significant effect on lactic acid production. The ranges of the different independent variables used were, respectively, 30-90 ml/l for the nitrogen source, 25-40°C for the temperature, and 300-800 g/l for the carbon source. A total of 20 experimental runs with six replications at the center point (Table 3) were completed, and evaluation of the lactic acid production expressed as response was determined. The result was mean of triplicate.

Data were fitted to a second-degree polynomial model (*Y*) of the form presented in the following:

(1) $$\begin{array}{c} Y=\beta_{0}+\beta_{1}X_{1}+\beta_{2}X_{2}+\beta_{3}X_{3}+\beta_{11}{X_{1}}^{2}+\beta_{22}{X_{2}}^{2}+\beta_{33}{X_{3}}^{2}+\beta_{12}X_{1}X_{2}+\beta_{13}X_{1}X_{3}+\beta_{23}X_{2}X_{3}, \end{array}$$

where *Y* represents the response (LA concentration); *β*_0_ is the constant; *β*_1_, *β*_2_, and *β*_3_ are the linear coefficients; *β*_11_, *β*_22_, and *β*_33_ are square coefficients; *β*_12_, *β*_13_, and *β*_23_ are the interaction coefficient; and *X*_1_, *X*_2_, *X*_3_, *X*_1_, *X*_22__,_*X*_32_, *X*_1_*X*_2_, *X*_1_*X*_3_, and *X*_2_*X*_3_ are the levels of independent variables. Three experimental replicates were performed under optimized conditions to validate the optimum conditions of LA production. The experimental and predicted concentration of LA were compared, and to verify the validity and robustness of the predictive model, the coefficient of determination (*R*^2^), bias factor (Bf), and absolute average deviation (AAD) were used.

### 2.8. Statistical Analysis

The Minitab 18 software was used for the experimental design; then the power of the model was assessed by evaluating the coefficient of determination (*R*^2^) obtained from the analysis of variance (ANOVA). Using the same software, the results expressed as a mean ± standard deviation were analyzed by the analysis of variance (ANOVA) test, followed by comparisons of the means between them by the Fisher test at the 0.05 probability threshold. Then, Sigma Plot v11.0 (c) systat was used for plotting the response surface plots.

## 3. Results

### 3.1. Lactic Acid Production

**Figure 1:**
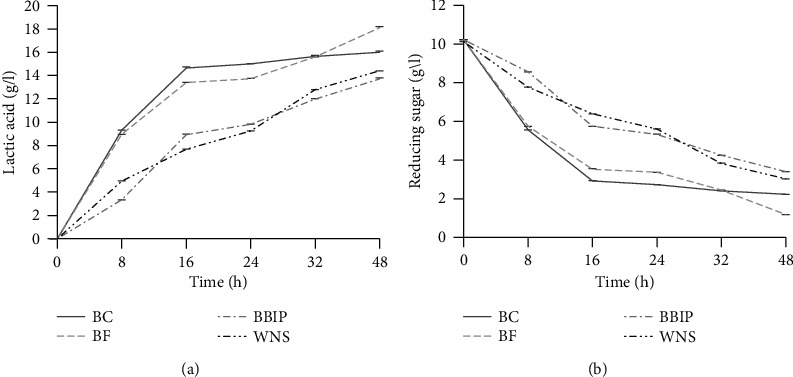
Kinetics of LA production (a) and reducing sugars consumption (b) in different production medium. BC: production medium containing by-products from chicken as nitrogen source; BIPB: production medium containing by-products of the beer production industry as nitrogen source; WNS: production medium without a nitrogen source; BF: production medium containing by-products from fish as nitrogen source.

The kinetics of LA production by *Lactiplantibacillus plantarum* strain 4O8 and those of the consumption of reducing sugars over time are illustrated in Figure 1. From this figure, it can be seen that LA production varies from one production medium to another; furthermore, there is a progression in LA concentrations over time and a subsequent decrease in reducing sugar concentrations in the different production media. In fact, in most fermentation broth, more than 50% of the initial quantity of reducing sugars is consumed by the fermenting strain between 0 and 16 hours of fermentation (Figure 1(b)), which results in a lower production of LA between 16 and 48 hours (Figure 1(a)). For this reason, the kinetic parameters (Table 4) were evaluated at 16 h after the start of fermentation. Carrying out fermentation in batch mode, the LA concentrations after 16 h of fermentation varied between 7.68 ± 0.00 g/l (fermentation broth without a nitrogen source (WNS)) and 14.64 ± 0.05 g/l (BC). Chicken by-products were the most nutritious source of nitrogen for the lactic ferment because, in this production medium, the volumetric productivity recorded (0.92 ± 0.00 g/l/h) was significantly different than the others (*p* < 0.05). Moreover, fish by-products came in the second position as they allowed the *Lactiplantibacillus plantarum* strain 4O8 to achieve a production performance in LA of 0.84 ± 0.00 g/l/h. Thus, it is clear from Table 4 that the best sources of nitrogen are BC and BF.

### 3.2. Determination of the Proximate Composition of the Best Nitrogen Sources

Table 5 shows the results obtained in the determination of the proximate composition of BC and BF. Majority of the parameters determined vary from one nitrogen source to another. Indeed, the highest contents of protein (83.00 ± 1.41%), phosphate (617.00 ± 2.83 mg/100 g), magnesium (58.54 ± 0.65 mg/100 g), and sodium (251.61 ± 0.56 mg/100 g) were present in BC; these values are significantly different from those obtained from BF (*p* < 0.05). The same applies to the potassium, iron, and zinc contents. However, BF contains the highest amounts of lipids (8.71 ± 0.13%), ash (6.00 ± 0.00%), and manganese (0.05 ± 0.00%); in fact, there is a significant difference between these contents and those obtained from the BC (*p* < 0.05). Moreover, no significant difference was observed between the dry matter and Ca contents of the two samples (*p* > 0.05).

Given the LA production obtained in the production medium containing BC as nitrogen source, and taking into account its protein content, it is clear that BC is the most promising nitrogen source.

### 3.3. Factors Influencing LA Production

Table 3 groups the experimental values of the responses measured during the experiments proposed by the experimental design according to the factors studied. It shows that the LA concentrations varied between 0.25 ± 0.02 g/l and 15.41 ± 0.05 g/l; this table also shows that the lowest LA concentrations were obtained when the incubation temperature was 45°C.

**Figure 2:**
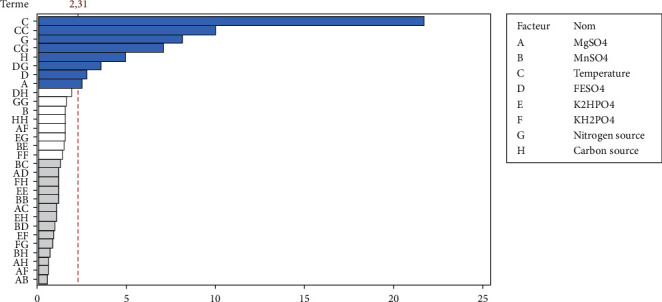
Pareto chart of the effects of variables on LA production in fermentation broth. The grey bars represent terms not included in the model.

*(1) ANOVA: Contribution of Factors*. The effects of factors on responses studied are illustrated by the Pareto diagram (Figure 2); it shows that among all the variables studied and at the chosen confidence level (*p* < 0.05), the linear and quadratic effects of temperature, nitrogen source, carbon source, FeSO_4_, and MgSO_4_ appear in decreasing order as very influential factors on LA production. Also, two interactions also showed a pronounced influence on the response studied; this was the interactions between temperature and the nitrogen source and the interaction between FeSO_4_ and the nitrogen source.

Table 6 presents the contributions of the most significant factors influencing (*p* < 0.05) LA production. It appears that the temperature is the factor having a major impact on the evaluated response (CF = 58.59 (*X*_3_) + 18.37 (*X*_3_*X*_3_) = 76.96) followed by the nitrogen source (CF = 8.20 (*X*_7_)) and carbon source (CF = 2.96 (*X*_8_)). The mathematical model (second-degree polynomial model) of relationship for LA with the most significant factors influencing LA production is given by the equation below. From this equation, it can be seen that the linear effects of temperature, nitrogen source, carbon source, FeSO_4_, and MgSO_4_ positively affect the evaluated response. In addition, the quadratic effect of temperature, and also the interactions between temperature and the nitrogen source and the one between FeSO_4_ and the nitrogen source negatively, affects the lactic acid production.

(2) $$\begin{array}{c} \begin{array}{c} Y=-110.3+1.693\text{ Mg}+6.560\text{ Temperature}+56.0\text{ FESO}4+0.3470\text{ Nitrogen source}\\ +0.00496\text{ Carbon source}-0.09251\text{ Temperature}\times \text{Temperature}\\ -0.007033\text{ Temperature}\times \text{Nitrogen source}-1.126\text{ FESO}4\times \text{Nitrogen source}. \end{array} \end{array}$$

### 3.4. Optimization of LA Production

**Figure 3:**
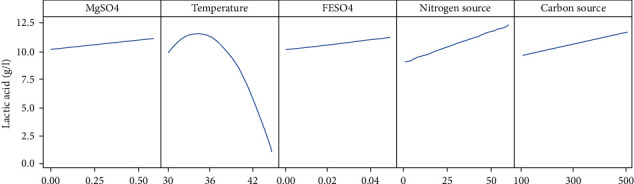
Effects of factors on LA production.

Here, temperature, carbon source, and nitrogen source were used for the optimization. Indeed, among the most significant factors influencing LA production, FeSO_4_ and MgSO_4_ have the lowest contributions on the evaluated response (0.93% and 0.77%, respectively). Furthermore, these factors have an insignificant contribution on the desirability of our model, which was 1. Moreover, the peak of the LA production was reached when the FeSO_4_ concentration was 0.00 g/l, and the curve translating the LA production for the MgSO_4_ interval considered was almost linear which expresses the absence of significant difference in LA production in this interval (Figure 3); it is for these reasons that these two factors were not taken into account for the optimization.

The LA concentrations obtained after the 20 experimental trials are summarized in Table 3. It appears that the LA concentrations ranged from 4.36 ± 0.02 g/l to 18.64 ± 0.01 g/l. Furthermore, this table also shows that the lowest LA concentration is obtained when the incubation temperature is the highest (44.75°C).

*(1) Contribution of independent variables and validation of the model*. The contribution of the independent variables and model validation are grouped in Table 7. It can be seen that the production of LA is influenced by linear and quadratic temperature effects, but also by the quadratic effects of the carbon source. Indeed, the temperature is the factor having the most effect on the production of LA because it has a major impact on the evaluated response (CF = 9.54 (*X*_2_) + 67.17 (*X*_2_*X*_2_) = 76.71). This table also shows that for the model, *R*^2^, AAD, and Bf were 92.98, 0.00, and 0.90, respectively. The mathematical equation resulting from our design of experiment optimization which can predict the variation of LA production according to the nitrogen source (*X*_1_), temperature (*X*_2_), and carbon source (*X*_3_) is mentioned in the following:

(3) $$\begin{array}{c} \begin{array}{c} Y=-20.96-0.1385\;X_{1}+3.076\;X_{2}-0.0207\;X_{3}+0.000690\;X_{1}\times X_{1}-0.05386\;X_{2}\times X_{2}\\ +0.000013\;X_{3}\times X_{3}+0.00201\;X_{1}\times X_{2}-0.000018\;X_{1}\times X_{3}+0.000273\;X_{2}\times X_{3}. \end{array} \end{array}$$

**Figure 4:**
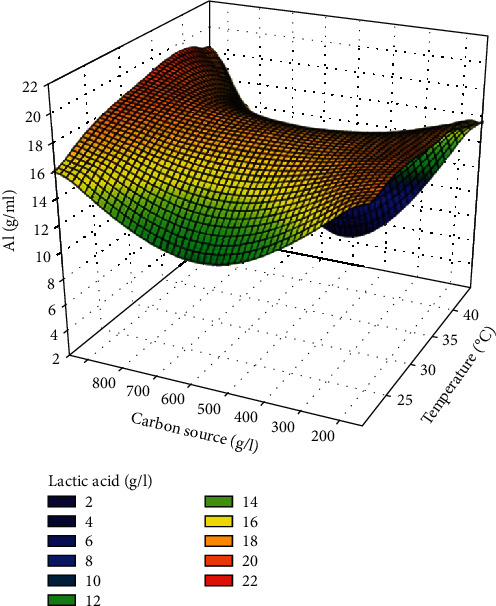
Response surface plot showing the interaction between carbon source and temperature on LA production.

*(2) Response Surface Plot of LA Production*. The interaction between temperature and carbon source was the one that showed a major impact on the evaluated response with a contribution of 1.11% (Table 7); it is for this reason that only the response surface plot showing the interaction between these two factors on LA production (Figure 4) was solicited. This figure shows that the areas of maximum activity are marked by the orange colour and correspond to LA concentrations between 20 and 22 g/l. The areas of minimum activity are represented by the blue colouring.

**Figure 5:**
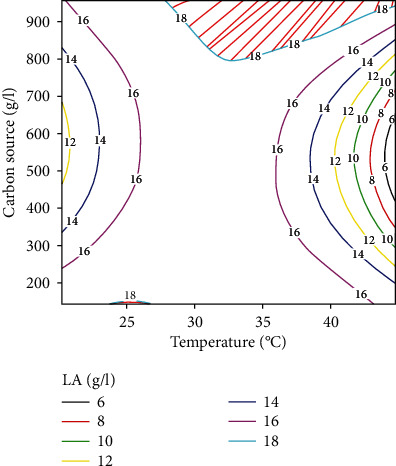
Contour plot showing the effect of carbon source and temperature on lactic acid production.

*(3) Contour Plot of LA Production*.  Figure 5 is a contour plot that visualises the areas of optimal variation of LA production as a function of temperature and carbon source content. The hatched areas represent the location of the maxima of this response, while the nonhatched areas represent the lowest values. The areas of interest are those where the LA concentration is higher than 18 g/l. These zones of interest define the experimental domain in which the application of the relevant conditions (temperature and carbon source content) will allow maximum LA production.

*(4) Optimum Conditions and Validation of the Optimal Conditions*. To confirm that there is coherence between theory and experiment optimum conditions, additional manipulations were carried out in the laboratory according to the optimal conditions predicted by the mathematical model (Table 8). The obtained experimental optimal values (20.93 ± 0.12 g/l) were compared to the predicted values (20.75 ± 00 g/l) to validate the optimal conditions defined by the model, and it was found that no significant difference was observed between these two values (*p* < 0.05).

### 3.5. LA Production Performance of *Lactiplantibacillus Plantarum* Strain 4O8

Table 9 summarises the LA production performance of *Lactiplantibacillus plantarum* strain 4O8 in the production medium that was not supplemented with nitrogen source, but also in the fermentation broth containing BC as nitrogen source with the nonoptimized conditions and under optimization conditions. From this table, it can be seen that LA concentration obtained in the production medium under optimizing conditions (20.93 ± 0.12 g/l) is significantly different than the others (*p* < 0.05). This table also shows that optimizing the production of LA using BC as a nitrogen source can multiply the performance of the lactic acid bacteria by a factor of 2.73.

## 4. Discussion

In our study, *Lactiplantibacillus plantarum* strain 4O8 showed its ability to produce significantly small amounts of LA after 16 h of fermentation in production media that were not supplemented with a nitrogen source. This phenomenon is explained by the low protein content of the pineapple by-products used as a carbon source for the preparation of the fermentation broth [8]. Indeed, to ensure good growth of the lactic ferment and consequent production of LA, the production medium must contain a sufficient quantity of protein to meet the nitrogen requirements of the fermenting strain [26]. Moreover, this LA production was improved when the different production media were supplemented with BC, BF, and BBIP; these by-products would therefore be potential sources of amino acids and peptides that served as a nitrogen source for the lactic ferment. Similar observations were reported by Kaktcham et al. [9] who in their work used fish by-products as a nitrogen source in a liquid medium formulated to optimize the production of bacteriocin by the *Lactococcus lactis* subsp. *lactis* 2MT strain. The low concentrations recorded in the various production media supplemented with BC, BF, and BBIP after 16 h of fermentation were due to the drop of more than 50% in the initial reducing sugar concentration, but also by the acidity of the medium due to the accumulation of LA [10], since the trials was performed in batch mode. The highest concentration of LA (14.64 ± 0.05 g/l) was obtained in the production medium in which BC was the nitrogen source; this can be explained simply by the protein composition of BC, which was the highest. These results are in agreement with those of Coelho et al. [4] who, in the context of their work, showed that LA production depended on the protein concentration in the production medium. The aforementioned LA concentration (14.64 ± 0.05 g/l) is significantly higher than that recorded (4.68 g/l) by Umesh and Preethi [13] who not only used pineapple by-products but also supplemented their production medium with MRS. The fact that fish by-products were the second most nutritious nitrogen source for *Lactiplantibacillus plantarum* strain 4O8 can be explained by its polyunsaturated fatty acid composition. Indeed Mouokeu et al. [27] showed in their work that omega-3 and omega-6 fatty acids (eicosapentaenoic and docosahexaenoic acid) had antimicrobial activity. Thus, these fatty acids would have been an obstacle to the optimal growth of the lactic ferment, resulting in a lower LA production.

BC and BF were the best nitrogen sources obtained in our investigation. The origin, genus, species, diet, geographical and climatic conditions, and the different parts of the animal constituting the by-products would be the plausible explanation for the differences between the values obtained when determining the proximate composition of these by-products [28–30].

Our study is also aimed at determining the factors that can influence the production of LA. Among the 8 factors considered, carbon source, nitrogen source (BC), temperature, MgSO_4_, and FeSO_4_ showed a major impact on the growth of *Lactiplantibacillus plantarum* strain 4O8 and consequently on its production of LA; similar data have been reported by several authors [4, 31, 32]. MnSO_4_, KH_2_PO_4_, and KHPO_4_ were the factors that in our study did not show a significant effect on the response studied (*p* > 0.05); these observations are contrary to those reported by Boudjelal and Nancib [32] and could be explained by the fact that the nutritional requirements of lactic acid bacteria in mineral salts vary from one strain of lactic acid bacteria to another to [33].

In the present study, temperature, carbon source, and nitrogen source were the factors used for optimization. Indeed, among the most significant factors influencing LA production, FeSO_4_ and MgSO_4_ had the lowest contributions on the studied response. Furthermore, these factors had an insignificant contribution to the desirability of our model. In addition, the peak of the LA production was reached when the FeSO_4_ concentration was 0.00 g/l, and the curve translating the LA production for the MgSO_4_ interval considered was almost linear which expresses the absence of significant difference in LA production in this interval. Thus, it is for these reasons that these two factors were not taken into account for the optimization. Similar data were reported by Myers et al. [34] whose work focused on response surface methodology: process and product optimization using designed experiment.

Within the framework of the optimization of the LA production, the values of the different mathematical tools for model validation obtained were 92.98, 0.00, and 0.9 for *R*^2^, AAD, and Bf, respectively. So, the *R*^2^ value indicates that 92.98% of the results obtained were attributed to the independent variables and only 7.02% of the variation cannot be explained by the model. Indeed, these values are in agreement with those reported in the literature (*R*^2^ > 0.75; AAD = 0; 0.75 < Bf < 1.25 for BF) [35], and therefore, our model accounts well for the variation in the response studied and can predict the evolution of our phenomenon.

The application of optimal conditions allowed us to obtain an experimental LA production of 20.93 ± 0.12 g/l (1.308 ± 0.01 g/l h): a value not significantly different from the optimal value predicted by the model which was 20.75 ± 0.00 (*p* > 0.05). It therefore emerges that to obtain maximum LA production after 16 hours of fermentation, a temperature of 30.89°C and a carbon and nitrogen source concentration of 141.75 g/l and 108.99 ml/l, respectively, are required. The optimal temperature mentioned above is in agreement with the data reported by Abbasiliasi et al. [31]; in fact, the *Lactiplantibacillus plantarum* strain 4O8 used as a fermentation agent in our study is a mesophilic lactic acid bacteria whose optimal growth temperature is between 30 and 40°C. The VP aforementioned (1.308 ± 0.01 g/l h) is higher than the one obtained (0.331 g/l h) by Mufidah and Wakayama [12] who, in the course of their work, used a lactic acid bacteria and a fungi strains for LA production from banana peel. Similarly, the most important LA productivity (1.12 g/l/h) reported by Costa et al. [36] in their study on lactic acid production from agri-food residues as carbon sources is lower than that highlighted in the present work. The use of carob, banana, and sugarcane lignocellulose biomass for LA production was the subject of a study conducted by Azaizeh et al. [37]; it revealed that LA production from these carbohydrate substrates with yeast extract were 54.8 g/l, 26.6 g/l, and 46.5 g/l, respectively. These LA concentrations are much important than the one obtained within the framework of our work. It was found that the production of LA would depend on nature and chemical composition of the carbohydrate and protein substrates in the fermentation broth and would also depend on the fermentation strain and fermentation conditions [3, 7, 10, 38].

In our study, the optimization of LA production led to an almost 1.43-fold (20.93 ± 0.12 g/l) increase compared with the nonoptimized conditions. This value is not far from the one obtained with the nonoptimized conditions (25.55 ± 0.04 g/l) in our previous studies (Ngouénam et al. [10]) in which we used not only the same lactic ferment but also the same carbon source; however, nitrogen source was yeast extract. Thus, the difference between these LA concentrations may be due to the richness of yeast extract in group B vitamins as reported by Gomez-Gomez et al. [39].

## 5. Conclusion

By-products from fish, chicken, and beer brewing industries showed their ability to boost the growth of *Lactiplantibacillus plantarum* strain 4O8 and consequently the production of LA. By-products from fish and chicken were the most nutritious sources of nitrogen for the fermentation agent; however, by-products from chicken had the highest protein content. Temperature, nitrogen source (products from chicken), carbon source were the factors that influence the production of LA in the best production medium especially the one in which the nitrogen source was chicken by-products. The optimization of the LA production in this medium allowed us to obtain an LA concentration of 20.93 ± 0.12 g/l. According to these results, chicken by-products are a potential nitrogen source that can be helpful in a large-scale production of LA. However, further studies should be oriented towards the use of these by-products for the lactic acid production in bioreactors, the purification of lactic acid present in the production medium, and the estimation of the percentage of lactic acid enantiomers.

## Figures and Tables

**Table 1:** Real levels of variables used in definitive plan.

Variables	Codes	Range and levels		
		-1	0	+1
MgSO_4_ (g/l)	*X*_1_	0	0.3	0.6
MnSO_4_ (g/l)	*X*_2_	0	0.025	0.05
Temperature (°C)	*X*_3_	30	37.5	45
FeSO_4_ (g/l)	*X*_4_	0	0.025	0.05
K_2_HPO_4_ (g/l)	*X*_5_	0	0.4	0.8
kH_2_PO_4_ (g/l)	*X*_6_	0	0.4	0.8
NS (ml/l)	*X*_7_	0	30	60
CS (g/l)	*X*_8_	100	300	500

**Table 2:** Definitive plan design (real and coded values) with the respective results.

No.	Independent variables								Lactic acid (g/l)	
	*X*_1_	*X*_2_	*X*_3_	*X*_4_	*X*_5_	*X*_6_	*X*_7_	*X*_8_	Experimental values	Predicted values
1	0.6 (1)	0.025 (0)	45 (1)	0 (-1)	0.8 (1)	0 (-1)	0 (-1)	500 (1)	*1.11 ± 0.01*	1.11
2	0 (-1)	0 (-1)	45 (1)	0 (-1)	0 (-1)	0.8 (1)	30 (0)	500 (1)	*1.11 ± 0.00*	1.05
3	0.6 (1)	0 (-1)	30 (-1)	0.05 (1)	0.4 (0)	0.8 (1)	0 (-1)	500 (1)	*9.41 ± 0.00*	9.59
4	0.6 (1)	0 (-1)	45 (1)	0.05 (1)	0 (-1)	0 (-1)	60 (0)	300 (0)	*1.13 ± 0.00*	1.39
5	0.3 (0)	0.025 (0)	37.5 (0)	0.025 (0)	0.4 (0)	0.4 (0)	30 (0)	300 (0)	*11.79 ± 0.00*	10.71
6	0 (-1)	0 (-1)	45 (1)	0.05 (1)	0.8 (1)	0.4 (0)	0 (-1)	100 (-1)	*0.90 ± 0.05*	0.95
7	0 (-1)	0.05 (1)	30 (-1)	0 (-1)	0.8 (1)	0.8 (1)	0 (-1)	300 (0)	*5.49 ± 0.01*	4.79
8	0 (-1)	0 (-1)	30 (-1)	0.025 (0)	0.8 (1)	0 (-1)	60 (1)	500 (1)	*12.94 ± 0.02*	13.65
9	0.6 (1)	0 (-1)	37.5 (0)	0 (-1)	0.8 (1)	0.8 (1)	60 (1)	100 (-1)	*11.48 ± 0.03*	12.19
10	0.6 (1)	0.05 (1)	30 (-1)	0 (-1)	0 (-1)	0.4 (0)	60 (1)	500 (1)	*15.41 ± 0.05*	14.96
11	0 (-1)	0.05 (1)	45 (1)	0 (-1)	0.4 (0)	0 (-1)	60 (1)	100 (-1)	*0.35 ± 0.02*	0.025
12	0.6 (1)	0.05 (1)	45 (1)	0.025 (0)	0 (-1)	0.8 (1)	0 (-1)	100 (-1)	*0.47 ± 0.02*	0.54
13	0.3 (0)	0.05 (1)	45 (1)	0.05 (1)	0.8 (1)	0.8 (1)	60 (1)	500 (1)	*1.88 ± 0.03*	1.88
14	0.3 (0)	0 (-1)	30 (-1)	0 (-1)	0 (-1)	0 (-1)	0 (-1)	100 (-1)	*3.35 ± 0.03*	4.31
15	0 (-1)	0.025 (0)	30 (-1)	0.05 (1)	0 (-1)	0.8 (1)	60 (1)	100 (-1)	*11.36 ± 0.00*	11.37
16	0.6 (1)	0.05 (1)	30 (-1)	0.05 (1)	0.8 (1)	0 (-1)	30 (0)	100 (-1)	*10.73 ± 0.02*	9.99
17	0 (-1)	0.05 (1)	37.5 (0)	0.05 (1)	0 (-1)	0 (-1)	0 (-1)	500 (1)	*10.58 ± 0.02*	10.94

(-1), (0), and (1) are coded levels.

**Table 3:** Experimental design for the optimization process of lactic acid production.

Run	Coded levels			Real levels			Lactic acid (g/l)	
	*X*_1_	*X*_2_	*X*_3_	Nitrogen source (ml/l) (*X*_1_)	Temperature (°C) (*X*_2_)	Carbone source (g/l) (*X*_3_)	Experimental values	Predicted values
1	1	-1	-1	90	25	300	*17.21 ± 0.00*	17.11
2	-1	-1	-1	30	25	300	*16.75 ± 0.01*	17.77
3	0	0	0	60	32.5	550	*17.60 ± 0.01*	17.64
4	1	-1	1	90	25	800	*15.52 ± 0.01*	16.32
5	0	0	0	60	32.5	550	*17.59 ± 0.01*	17.64
6	-1	1	-1	30	40	300	*14.65 ± 0.02*	13.52
7	-1	-1	1	30	25	800	*17.22 ± 0.02*	17.53
8	1	1	1	90	40	800	*17.27 ± 0.01*	15.93
9	0	0	0	60	32.5	550	*16.95 ± 0.02*	17.64
10	0	0	0	60	32.5	550	**17.10 ± 0.00**	17.64
11	-1	1	1	30	40	800	*15.55 ± 0.02*	15.33
12	1	1	-1	90	40	300	*15.30 ± 0.03*	14.66
13	0	0	-1.633	60	32.5	141.75	*17.55 ± 0.00*	17.92
14	0	0	0	60	32.5	550	*17.00 ± 0.00*	16.22
15	0	0	0	60	32.5	550	*16.90 ± 0.00*	16.22
16	0	-1.633	0	60	20.25	550	*11.43 ± 0.03*	10.03
17	0	1.633	0	60	44.75	550	*4.36 ± 0.02*	6.24
18	1.633	0	0	109	32.5	550	*17.22 ± 0.02*	17.85
19	0	0	1.633	60	32.5	958.25	*18.64 ± 0.01*	18.76
20	-1.633	0	0	11.01	32.5	550	*18.04 ± 0.01*	17.89

*X*_1_: nitrogen source; *X*_2_: temperature; *X*_3_: carbon source.

**Table 4:** Lactic acid, volumetric productivity, and lactic acid yield after 16 h of fermentation.

Nitrogen sources	Lactic acid concentration (g/l)	Volumetric productivity (g/l h)	Lactic acid yield (mg/g)
BC	14.64 ± 0.05^a^	0.92 ± 0.00^a^	29.80 ± 0.14^a^
BF	13.4 ± 0.02^b^	0.84 ± 0.00^b^	26.91 ± 0.13^b^
BBIP	8.23 ± 0.04^c^	0.56 ± 0.00^c^	17.80 ± 0.00^c^
WNS	7.68 ± 0.00^d^	0.48 ± 0.00^d^	15.63 ± 0.00^d^

^a,b,c,d^Values with different letters on the same column differ significantly each other (*p* < 0.05).

**Table 5:** Proximate composition of BC and BF.

Parameter	BC	BF
Dry matter (% DM)	91.71 ± 3.32^a^	92.78 ± 2.32^a^
Fat (% DM)	1.41 ± 0.11^b^	8.71 ± 0.13^a^
Protein (% DM)	83.00 ± 1.41^a^	74.00 ± 1.41^b^
Ash (% DM)	4.00 ± 0.00^b^	6.00 ± 0.00^a^
P (mg/100 g)	617.00 ± 2.83^a^	601.50 ± 2.12^b^
Ca (mg/100 g)	743.50 ± 0.71^a^	741.00 ± 1.41^a^
Mn (mg/100 g)	0.00 ± 0.00^b^	0.05 ± 0.00^a^
Mg (mg/100 g)	58.54 ± 0.65^a^	55.83 ± 0.240^b^
Na (mg/100 g)	251.61 ± 0.56^b^	302.66 ± 0.484^a^
K (mg/100 g)	510.00 ± 1.41^a^	503.00 ± 1.41^b^
Fe (mg/100 g)	57.42 ± 0.12^a^	49.62 ± 0.23^b^
Zn (mg/100 g)	1.5 ± 0.014^a^	0.87 ± 0.07^b^

^a,b^Values with different letters on the same column differ significantly each other (*p* < 0.05). BC: by-product from chicken; BF: by-product from fish.

**Table 6:** Analysis of variance (*p* values, contribution of factors (CF), and coefficient of factors (Coeff).

	Coeff	*p*	CF%
*X*_1_	0.508	0.0370	0.77
*X*_3_	-4.417	0.0001	58.59
*X*_4_	0.556	0.0250	0.93
*X*_7_	1.653	0.0001	8.20
*X*_8_	0.993	0.0010	2.96
*X*_3_ *X*_3_	-5.204	0.0001	18.37
*X*_3_ *X*_7_	-1.583	0.0001	7.64
*X*_4_ *X*_7_	-0.845	0.0080	1.54

*X*_1_: MgSO_4_; *X*_3_: temperature; X_4_: FeSO_4_; X_7_: nitrogen source; X_8_: carbon source.

**Table 7:** *p* values, contribution of factors (CF) coefficient of determination (*R*^2^), absolute average deviation (AAD), and bias factor (Bf) for optimized production of LA following CCD design.

	*p*	CF%
*X*_1_	0.9630	0.00
*X*_2_	0.0070^∗^	9.54
*X*_3_	0.4580	0.47
*X*_1_ *X*_1_	0.0960	4.20
*X*_2_ *X*_2_	0.0001^∗^	67.17
*X*_3_ *X*_3_	0.0410^∗^	4.43
*X*_1_ *X*_2_	0.3200	0.87
*X*_1_ *X*_3_	0.7580	0.08
*X*_2_ *X*_3_	0.2630	1.11
Model validation		
*R* ^2^		92.98
AAD		0.00
Bf		0.9

^∗^Independent variable that significantly (*p* < 0.05) affects the response. *X*_1_: nitrogen source; *X*_2_: temperature; *X*_3_: carbon source.

**Table 8:** Predicted and experimental lactic acid production using optimal culture conditions.

Parameters	Values
Optimum conditions	
Nitrogen source (ml/l)	108.99
Temperature (°C)	30.89
Carbon source (g/l)	141.75
Validation of optimum conditions	
Optimal predicted concentration of LA (g/l)	20.75 ± 00^a^
Optimal experimental concentration of LA (g/l)	20.93 ± 0.12^a^
Desirability	1

^a^Values with the same letter on the same column are not significantly different each other (*p* < 0.05).

**Table 9:** Lactic acid production of *Lactiplantibacillus plantarum* strain 4O8 using BC and yeast extract.

Production medium	PM-WNS	PM-BC without optimization	PM-BC under optimization conditions
Lactic acid (g/l)	7.68 ± 0.00^c^	14.64 ± 0.05^b^	20.93 ± 0.12^a^
Increase coefficient	*X*	1.91*X*	2.73*X*

^a,b,c,d^Values with different letters on the same column differ significantly each other (*p* < 0.05). PM: production medium; PM-WNS: production medium without nitrogen source; PM-BC: production medium containing by-product from chicken as nitrogen source.

## Data Availability

No funding was obtained for this work.
